# Editorial Note: Marked reduction in fertility among African women with urogenital infections: A prospective cohort study

**DOI:** 10.1371/journal.pone.0338993

**Published:** 2025-12-18

**Authors:** The PLOS One Editors

The *PLOS One* Editors issue this Editorial Note because this article [1] was identified as one of a series of submissions for which we have concerns about aspects of the peer review process. Readers are advised to interpret the article [1] with caution.

The Editors have no evidence of any author involvement in the peer review process.
